# Chitosan-Based Beads Incorporating Inorganic–Organic Composites for Copper Ion Retention in Aqueous Solutions

**DOI:** 10.3390/ijms25042411

**Published:** 2024-02-18

**Authors:** Andreea Miron, Tanta-Verona Iordache, Artur J. M. Valente, Luisa Maria Rocha Durães, Andrei Sarbu, Georgeta Ramona Ivan, Anamaria Zaharia, Teodor Sandu, Horia Iovu, Anita-Laura Chiriac

**Affiliations:** 1 Advanced Polymer Materials and Polymer Recycling Group, National Institute for Research & Development in Chemistry and Petrochemistry ICECHIM, Spl. Independentei 202, 6th District, 060021 Bucharest, Romania; andreea.miron@icechim.ro (A.M.); tanta-verona.iordache@icechim.ro (T.-V.I.); andrei.sarbu@icechim.ro (A.S.); georgeta.ivan@icechim.ro (G.R.I.); anamaria.zaharia@icechim.ro (A.Z.); teodor.sandu@icechim.ro (T.S.); 2 Advanced Polymer Materials Group, National University of Science and Technology Politehnica Bucharest, 1–7 Gh. Polizu Street, 011061 Bucharest, Romania; horia.iovu@upb.ro; 3 CQC-IMS, Department of Chemistry, University of Coimbra, Rua Larga, 3004-535 Coimbra, Portugal; avalente@ci.uc.pt; 4 CIEPQPF, Department of Chemical Engineering, University of Coimbra, Rua Sílvio Lima, 3030-790 Coimbra, Portugal; luisa@eq.uc.pt

**Keywords:** chitosan, inorganic–organic composites, polymeric beads adsorbents, water treatment, Cu^2+^ removal

## Abstract

In recent years, there has been a challenging interest in developing low-cost biopolymeric materials for wastewater treatment. In the present work, new adsorbents, based on different types of chitosan (commercial, commercial chitin-derived chitosan and chitosan synthesized from shrimp shell waste) and inorganic–organic composites have been evaluated for copper ions removal. The efficacy of the synthesis of chitosan-based composite beads has been determined by studying various characteristics using several techniques, including FTIR spectroscopy, X-ray diffraction, porosimetry (N_2_ adsorption), and scanning electron microscopy (SEM). Adsorption kinetics was performed using different adsorption models to determine the adsorption behavior of the materials in the aqueous media. For all composite beads, regardless of the type of chitosan used, good capacity to remove copper ions from simulated waters was observed (up to 17 mg/g), which proves that the new materials hold potential for heavy metal retention. However, the adsorption efficiency was influenced by the type of chitosan used. Thus, for the series where commercial chitosan (CC) was used, the removal efficiency was approximately 29%; for the series with chitosan obtained from commercial chitin (SC), the removal efficiency was approximately 34%; for the series with chitosan enriched with CaCO_3_ (SH), the removal efficiency was approximately 52%.

## 1. Introduction

Pollution, in general terms, is a process of introducing toxic substances into the environment, causing devastating effects on ecosystems. In general, pollutants can be classified into several categories—chemical, biological, and natural chemical—and are derived from several sources such as anthropogenic activities, hazardous waste generation, or industrial emissions. Pollution is a factor that has a negative impact on the quality of water sources as well as air and soil. Water is an extremely important resource for sustaining life on Earth [1,2]. Due to increasing population density, industrial development and technology, water pollution has become a serious global problem. In this context, the rapid expansion of communities and industrial development near waterways has led to increased environmental hazards, especially due to heavy metal pollution. Therefore, contamination of water sources, with concentrations above the maximum limits allowed under the legislation, is mainly due to agricultural activities but also industrial activities (mining, metallurgy, refineries, etc.). Among the most harmful heavy metals, Cd^2+^, Zn^2+^, Cr^3+^, Ni^2+^, Pb^2+^ and Mn^2+^ can be mentioned [2,3]. The legislation lays down maximum limits for the discharge of wastewater into the aquatic environment, but these limits are often exceeded due to inefficient treatment of water from industrial activities [4]. In addition to anthropogenic activities (industry, mining, agriculture, etc.), natural processes such as volcanic eruptions, grinding of rocks with metal content, and forest fires are also responsible for contaminating the aquatic and terrestrial ecosystem with heavy metals [5]. The major problem of heavy metal contamination is the multiple routes through which such pollutants enter the human body, i.e., direct ingestion, skin absorption, and inhalation through the mouth and nose [6]. From water, heavy metals most often enter the human body through ingestion or dermal absorption [7]. 

Among the main techniques used to remove heavy metal ions from wastewater, we can mention ion exchange [8], membrane filtration techniques [9], chemical precipitation [10], electrochemical treatment technology, biological treatment, and adsorption [11,12]. Nevertheless, all these techniques have advantages and disadvantages; the most notable disadvantages are high production costs, generation of potentially hazardous waste, and high energy costs [11]. Adsorption using adsorbent materials can overtake some of those drawbacks. Adsorption is one of the most common treatments widely applied in industry to remove heavy metals from water. Recent studies have shown a great interest of researchers in the production of inexpensive and environmentally friendly adsorbent materials. Several classes of adsorbents are presented in the literature that are effectively used to remove heavy metals from water [13,14]. Among them, commercially available adsorbents such as graphene and carbon nanotubes can be mentioned [15]. Yet, their high adsorption properties do not seem to balance their high price. Thus, farther research was directed towards the production of more cost-effective adsorbent materials ranging from natural adsorbents such as zeolites, titanium dioxide, or silica, to waste-derived adsorbents (using eggshells, fruit/vegetable peels, shellfish carcasses but also industrial waste such as red mud and ash [16]).

Inorganic structures such as silica, zeolites, titania, alumina, and carbon derivatives (carbon nanotubes, activated carbon, graphene oxide) have also been used as adsorbents due to their properties such as thermal and mechanical stability, porosity, the orderly structure that inorganic structures generally possess and the relatively easy to adjust size and shape [17]. In addition, their abundance in nature together with the recent industrial development has led to the wide use of these inorganic structures as adsorbents [18]. On the other hand, the major problem with the use of inorganic materials is the disposal of waste, generally requiring the use of chemicals and generating hazardous waste that is dangerous to the environment [19]. Yet, recent studies have shown that the use of composites instead of the inorganic material alone is more efficient and may reduce the amount of waste after treatment, especially because the waste materials can be collected and reconditioned for reuse [20,21,22,23]. Other notable advantages of using composites are given by the increased surface area, resulting in higher affinity or selectivity for a target ion, and improved textural characteristics and mechanical properties, such as elasticity [24]. 

In recent years, chitosan (CC), a chitin-derived polysaccharide, has been reported as a promising biopolymer for the development of adsorbents due to its properties such as biodegradability, low price and abundance in nature [25,26]. Chitosan is obtained most often by the chemical deacetylation of chitin, this being found in the exoskeleton of marine crustaceans such as shrimps, crayfish, lobsters, and crabs. Therefore, the cheapest way to obtain chitosan is by recovering waste carcasses from the food industry. This action can have a double environmental effect: (1) reuse of waste to obtain new materials; and (2) use of the materials to treat wastewater [27]. Furthermore, the literature has provided information about the ability of chitosan alone to remove a wide variety of heavy metals such as cadmium, nickel, copper, and chromium [28]. This is mainly due to hydroxyl and amino groups occurring in the chitosan structure, available to form strong coordination bonds with metal ions. However, biopolymers such as chitosan have their limitations in terms of thermal and mechanical resistance. Therefore, it is mandatory to combine biopolymers with other materials that can improve the targeted properties of the final adsorbent [29,30]. In this respect, inorganic materials are some of the most used reinforcing components for improving the features of thus formed composites [24]. For instance, Hasan et al. [31] obtained polymer composite beads based on chitosan and perlite for the removal of Cu^2+^ from wastewater. Their study has demonstrated that the adsorption capacity for Cu^2+^ was approximately 104 mg/g, at pH 4.5. 

The interest in developing new materials capable of absorbing copper has been driven by the fact that copper is a valuable and widely used metal [16,32], but, at the same time, when present in the environment in excessive amounts, it can have harmful effects on ecosystems and human health [33]. Long-term exposure of the human body to high concentrations of copper has been associated with adverse health effects including gastrointestinal disorders, tachycardia, respiratory problems, insomnia [34]. As a result, the European Union, through the World Health Organization (WHO), has enforced the maximum acceptable limit of copper in drinking water at 2 mg/L. Thus, industries should explore new and efficient methods of treating the effluents resulting from their activities in order to meet these legislative requirements [2,16]. In this context, the goal of the present paper was to develop polymeric beads based on chitosan and inorganic–organic composites for advanced treatment of waters containing heavy metal ions, with particular focus on Cu^2+^ removal. In an original attempt, the beads prepared in this study were obtained with a titanium oxide–polyacrylonitrile inorganic–organic composite, prepared by a host–guest method [35] for obtaining intermediates for titanium nitride. The use of this type of filler material for preparing chitosan-based beads as adsorbent materials for Cu^2+^ is described herein for the first time. The samples were first characterized to determine the influence of the chitosan type and the filler percentage on the structure, morphology, specific surface area, pore size and volume. However, the following adsorption assays helped clarify the mechanism for Cu^2+^ retention by fitting the data to several kinetic adsorption models.

## 2. Results and Discussion 

### 2.1. Synthesis of Composite Beads

For a better understanding of the influence of chitosan type and filler percentage on bead properties, studies were performed using three types of chitosan (commercial chitosan—CC, chitosan obtained from commercial chitin—SC and chitosan obtained from shrimp carcasses waste containing native minerals—SH) with different inorganic–organic composite ratios (10% and 33%, respectively). The samples are designated as X-Ti_PAN Y% where X represents the type of chitosan used (CC, SC, or SH) and Y the percentage of the titanium oxide–polyacrylonitrile composite filler.

#### 2.1.1. Structure Evaluation of Composite Beads 

**Figure 1:**
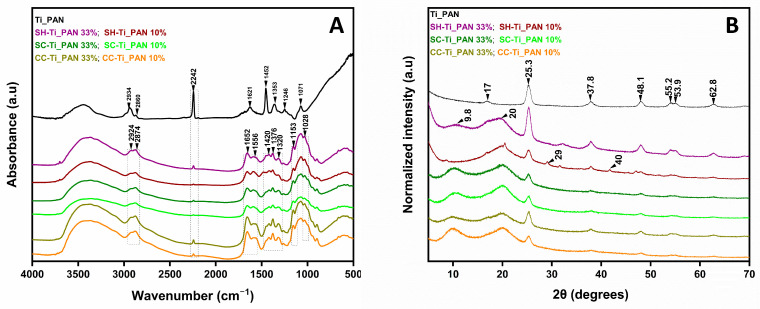
Structure evaluation by FTIR (**A**) and XRD (**B**), for the chitosan-based polymeric (CC-Ti_PAN 10%, CC-Ti_PAN 33%, SC-Ti_PAN 10%, SC-Ti_PAN 33%, SH-Ti_PAN 10% and SH-Ti_PAN 33%) beads compared to Ti_PAN composite reference.

To emphasize the distinct groups in the chitosan-based beads, Figure 1a compares the FTIR spectra of the three series of beads with that of titanium oxide- polyacrylonitrile filler alone. Studies involving beads with different filler ratios were carried out and several similarities between the beads were observed. 

According to the literature, the typical bands for the functional groups of chitosan are observed at 2924–2874 cm^−1^, associated with the stretching vibration of the -CH bond, which is a characteristic bond in polysaccharides including other species such as xylan and glucan [20]. The bands at 1320 cm^−1^, assigned to the C=O tensile vibration in amide III, and at 1652 cm^−1^, for the C=O bond stretching vibration in amide I, are characteristic and distinct for residual N-acetyl groups [36]. The low intensity of the band at 1556 cm^−1^, which is characteristic of the N-H group bending vibration in amide II, may be justified by the higher deacetylation degrees of all chitosan samples used in this study [37]. In addition, chitosan exhibits bands at 1420 cm^−1^ and 1376 cm^−1^, characteristic of symmetric deformations of the -CH_2_ and -CH_3_ groups, respectively, while the characteristic bands of -NH oscillations and C-O-C extension (indicates the saccharide structure of chitosan) are present in the spectra at 1153 cm^−1^ and 1028 cm^−1^, respectively [38]. 

C-H vibrations may be observed at 1246, 1353, and 1452 cm^−1^ in the Ti_PAN composite reference spectrum, as well as in all the spectra of beads. A band at 1455 cm^−1^ suggests CH2 deformation, while the methylene group’s stretching vibrations are displayed at 2860 and 2934 cm^−1^. At the same time, the stretching vibration of the C≡N group, characteristic of polyacrylonitrile, can be observed at 2242 cm^−1^, which confirms the presence of the composite filler in the polymeric beads [35,39]. 

XRD patterns of the all-polymeric beads and the composite (Figure 1b) show strong diffraction peaks at 2θ values of 25.3°, 37.8° and 48.1°, indicating TiO_2_ in the anatase phase corresponding to planes 101, 103 and 004 [31]. In addition, the composite shows a characteristic peak of acrylonitrile approximately 17.0° attributed to the (100) plane of pure polymer [40]. In the case of polymeric beads this peak most likely disappears due to the high peak intensity at 20°. At the same time, other peaks, with lower intensity, confirm the presence of the anatase phase at 2θ values of 53.9°, 55.2°, 62.8° corresponding to the planes 105, 211 and 118 [41]. Thus, the success of the composite filler incorporation into the chitosan-based beads is confirmed. In agreement with the previous work [42], chitosan shows two characteristic peaks at 2θ values of 9.8° and 20° corresponding to the 020 and 110 planes observed in all types of chitosan-based beads, which indicate the presence of ordered crystalline structure of chitosan. In the SH-Ti_PAN 10% sample, the characteristic peaks for CaCO_3_ can be observed at 2θ values of 29° and 40° attesting its presence from the initial synthesis of chitosan [43]. Their absence in the SH-Ti_PAN 33% beads may be attributed to its lower ratio in the composite as a result to the increased amount of the inorganic–organic composite, particularly of TiO_2_. Other differences may be observed, such as disappearance of the chitosan peak at 9.8° in SH-Ti_PAN 10% and similar peak intensities of TiO_2_ regardless of Ti_PAN content for SC-based systems, which may be due to the heterogenous distribution of chitosan and TiO_2_ in the material.

#### 2.1.2. N_2_ Adsorption/Desorption Measurements and Morphology of Composite Beads

**Figure 2:**
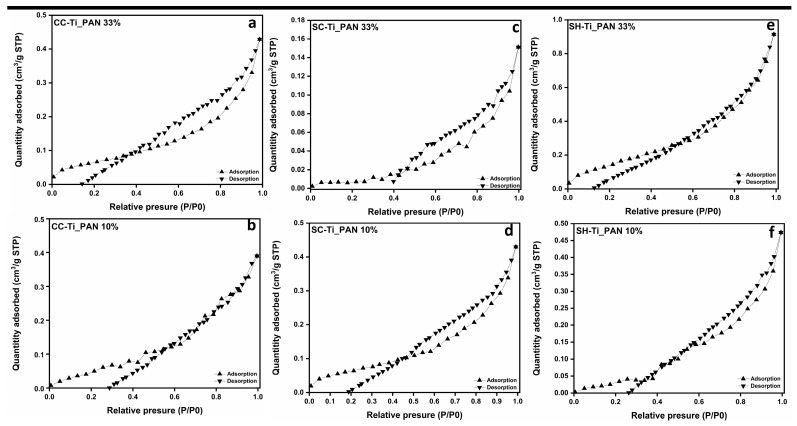
Adsorption/desorption isotherms and pore size distribution for series of chitosan-based polymer (CC-Ti_PAN 10% (**a**); CC-Ti_PAN 33% (**b**); SC-Ti_PAN 10% (**c**); SC-Ti_PAN 33% (**d**); SH-Ti_PAN 10% (**e**); SH-Ti_PAN 33% (**f**)) samples.

The measurement of N_2_ adsorption/desorption was carried out to reveal the specific surface area and porosity of the composite beads. Figure 2 shows the isotherms and pore size distribution for each type of composite beads. In agreement with the IUPAC nomenclature, which classifies BET isotherms into six types [44], the polymer beads presented herein reveal a type IV curve alignment, specific to mesoporous materials. The hysteresis loop presented in each case is H-4 type, with an almost horizontal and parallel aspect over a wide pressure (P/P0) range. This type of hysteresis loop is often associated with narrow pores [42].

**Figure 3:**
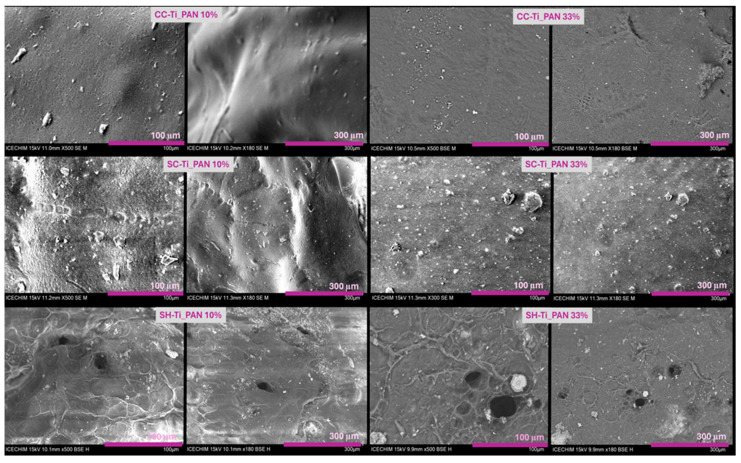
SEM micrographs for the composite beads series based on commercial chitosan (CC-Ti_PAN 10%; CC-Ti_PAN 33%, commercial chitin-derived chitosan (SC-Ti_PAN 10%; SC-Ti_PAN 33%) and chitosan obtained from shrimp wastes (SH-Ti_PAN 10%; SH-Ti_PAN 33%) at 100 µm and 300 µm.

Furthermore, the BET surface area and pore surface area for each type of polymeric beads, as resumed in Table 1, present important variations with the change in chitosan type or with the increase in filler. Interestingly, all the beads have presented little variation in pore diameter and volume, which means that the filler has a very low influence on the porosity of the composite beads, regardless of the amount used. The largest BET surface area (3.334 m^2^g^−1^) was recorded for SH-Ti_PAN 10%, which also revealed the largest pore surface area (9.443 m^2^g^−1^) and micropore volume (0.011 cm^3^g^−1^). The obtained data from BET were also confirmed by the morphology analysis in Figure 3, which in the case of SH-based beads, meaning SH-Ti_PAN 10% and SH-Ti_PAN 33%, Figure 3e,f, respectively, displays the presence of large pores on the surface of the beads. Meanwhile, CC-Ti_PAN 33% and SC-Ti_PAN 33% beads (Figure 3b,d, respectively) present a homogeneous but rough surface with no visible pore structures. Yet, the presence of the titanium oxide–polyacrylonitrile filler is visible on the surface of all beads, as granular microstructures of different sizes dispersed relatively homogeneously.

### 2.2. Adsorption Kinetics of Cu^2+^ Ions

In this section, three important factors that characterize the adsorption processes (in terms of adsorption capacity, efficiency, and adsorption mechanism) were investigated and described. 

**Figure 4:**
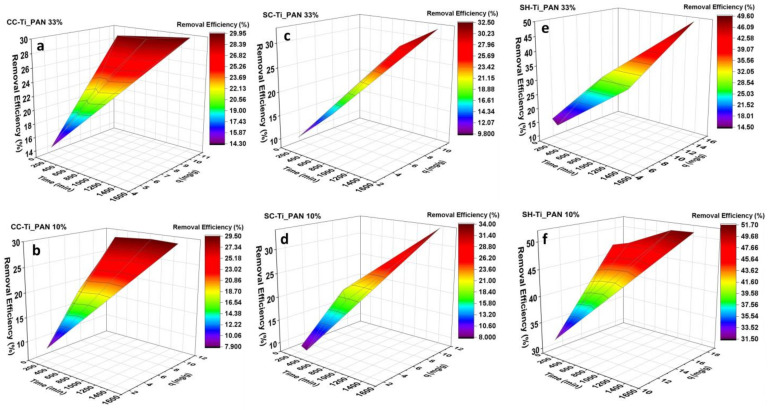
Effect of time on the removal efficiency and adsorption capacity on Cu^2+^ adsorption at 25 °C, for the CC series (**a**,**b**); SC series (**c**,**d**) and SH series (**e**,**f**) (in this experiment R_S/L_ = 1.5 mg/mL; pH = 5 and initial concentration of 100 mg/L).

In this respect, Cu^2+^ adsorption at pH = 5 and 25 °C was measured as a function of contact time starting from a solution of 100 mg/L, and R_S/L_ = 1.5 mg mg/L, as shown in Figure 4. Adsorption studies using chitosan-based composite beads were performed over a time range from 90 min (1.5 h) to 1440 min (24 h). The adsorption rate was quite fast in the first 420 min, when attaining equilibrium and forming a plateau. It can be observed that both the percentage of titanium oxide–polyacrylonitrile filler and the type of chitosan influence the Cu^2+^ retention properties onto polymeric beads. A clear difference can be noticed for the series of beads with SH (Figure 4c and Table 2—adsorption capacities), in which case the polymeric beads with lower amount of composite, i.e., SH-Ti_PAN 10%, retain higher amounts of Cu^2+^ than the counterpart with 33% composite (up to 17.03 mg/g vs. 15.52 mg/g, respectively). However, for the series with CC and SC, lower adsorption capacities were registered at 10% composite amount vs. 33%. It can also be noted that the highest adsorption capacity was attained by the SH-Ti_PAN 10% system. The removal efficiency (%) of the polymeric beads for Cu^2+^ was also calculated and summarized in Table 2, in which case it can be observed that the highest Cu^2+^ retention efficiency is again recorded for the system SH-Ti_PAN 10% (51.7%). It can also be observed that removal efficiency depends on the type of chitosan used and the percentage of inorganic–organic composite present in the structure of the polymer composite beads, and ranges from 29.5% and 51.6%. Thus, for commercial chitosan-based beads, the removal efficiency (Figure 4) is similar for both filler percentages (approximately 30%); for beads obtained from commercial chitin-derived chitosan, it is approximately 33% and for samples with chitosan obtained from shrimp shell waste, it is 49.6% for 33% filler and 51.6% for 10% filler, respectively.

To evaluate the improvement for Cu^2+^ adsorption properties recorded for the polymer beads; the data obtained herein were compared with other literature records for several types of adsorbents. Table 2 provides a comparison between the adsorption capacity of polymer beads and adsorbent materials obtained by other authors [45,46,47], where it can be observed that the adsorption capacity of the polymeric beads is significantly higher compared to references [46,47]. However, in the study of Wan et al. [44], the high removal efficiency of 99.77% is also due to a high R_S/L_ value, of approximately 83.33 mg/mL (2.5 g of sample in 30 mL of solution) compared to the present study, in which case the R_S/L_ was 1.5 mg/mL (meaning 15 mg of sample to 10 mL of Cu^2+^ ion solution). For the other two studies [46,47], the removal efficiency was not reported.

**Figure 5:**
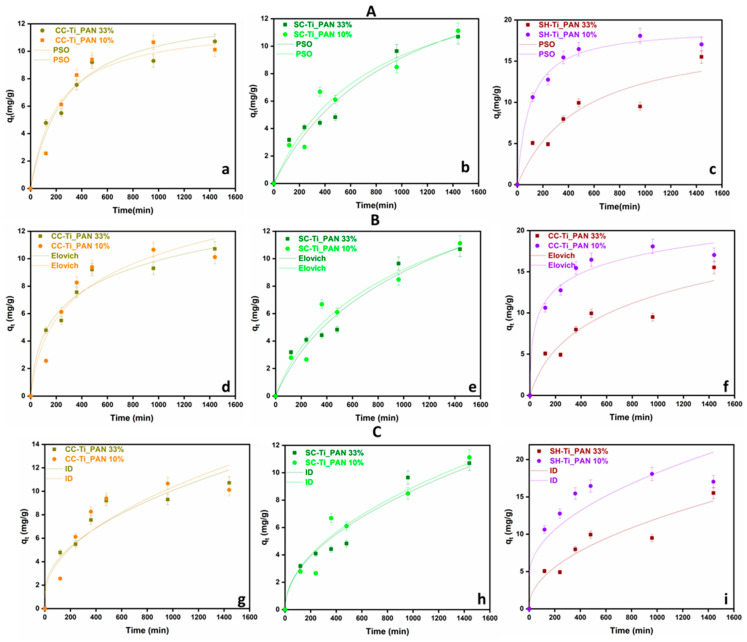
Kinetic models PSO (**A**), Elovich (**B**) and ID (**C**) for Cu^2+^ ion adsorption for chitosan-based polymer beads at R_S/L_ = 1.5 mg/mL, pH = 5, initial Cu^2+^ concentration of 100 mg/L, time range 0–1440 min, and error bars of 5% relative to the measured raw data, for the CC series (**a**,**d**,**g**); SC series (**b**,**e**,**h**) and SH series (**c**,**f**,**i**).

Further on, the assessment of adsorption kinetics has been used to ascertain the reaction rate and the adsorption mechanism. The mechanism of adsorption kinetics is an important parameter, as it describes the adsorption rate of the adsorbent and controls the residual time of the whole process. To better understand the adsorption process, the pseudo-second-order kinetic (PSO), Elovich and intraparticle diffusion (ID) (Figure 5) models were fitted to the existing experimental data and the parameters are summarized in Table 3. The determination coefficient (R^2^) for PSO ranged from 0.898 to 0.989, for Elovich values ranging from 0.910 to 0.975 and for ID between 0.772 and 0.958.

The best representation of the results overall came from the PSO model, followed by Elovich and ID. The PSO model suggests surface-limited adsorption, which is explained by the formation of metal–ligand bonds [48,49]. It can also be mentioned that SC-Ti_PAN 10% and SH-Ti_PAN 33% samples were better fitted by the Elovich model, indicating that, for these two types of materials, the adsorbing surfaces are more heterogeneous and this influences the chemisorption mechanism in a higher extent [50]. Moreover, it seems that all beads’ systems are limited by a chemisorption mechanism, since the ID model (that describes adsorption processes through diffusional mechanisms) was not very well adjusted on the experimental data. 

Therefore, the PSO kinetic model revealed that CC-Ti_PAN 33% beads show a moderate equilibrium adsorption (*q_e_*) and a moderate adsorption rate (k_2_). However, for the CC-Ti_PAN 10%, the *q_e_* value is slightly higher and the adsorption rate slightly slower. Polymeric beads SC-Ti_PAN 33% and SC-Ti_PAN 10% also show good *q_e_* values while k_2_ indicates a relatively fast adsorption rate compared to the other studied samples. For the shrimp waste-derived chitosan samples (SH-Ti_PAN) the behavior is relatively similar. In the case of the SH-Ti_PAN 10%, the *q_e_* value is high and the velocity, represented by the k_2_ value shows a slower adsorption rate. However, the R^2^ value is the lowest, suggesting a lower accuracy of the PSO kinetic model compared to the other studied models. On the other hand, the SH-Ti_PAN 10% sample shows the highest equilibrium concentration value and a moderate to high k_2_ rate, with a good fitting of the pseudo second-order kinetic model.

For the Elovich kinetic model, the *R^2^* values in the range of 0.910–975 suggest a fairly good fit of the model to all the experimental data. The desorption constant *β* values range from 0.167 to 0.385, suggesting that the interaction between the adsorbent molecules and the adsorbent surface is relatively similar regardless of the sample, while *α* (initial adsorption rate) has a significant variation between samples. The 1.196 maximum value of α was found for the SH-Ti_PAN 10% sample, indicating a higher adsorption rate and a higher affinity of the beads for Cu^2+^. At the same time, the lowest α value was determined for the SC-Ti_PAN 33% sample, indicating a lower affinity of the beads for Cu^2+^ [51,52,53].

The intraparticle diffusion kinetic model reveals *k_dif_* values in the range 0.275–0.437 mg/g min^1/2^, indicating that the diffusion velocity has the same order of magnitude. However, the value of *C* has a significant variation between the samples, i.e., 0.925–9.900 mg/g, suggesting major differences in the variation in the boundary layer. The highest values of *C* recorded for the SC series, meaning 9.900 mg/g for SC-Ti_PAN 33% and 9.890 mg/g SC-Ti_PAN 10%, indicate a faster rate of intraparticle diffusion compared to the other chitosan types [54,55,56]. 

In conclusion, the kinetic study indicated that the adsorption properties are more linked to the porous structure of beads. Nevertheless, the higher deacetylation degree of SH (which means more -NH_2_ groups that bind Cu^2+^) may also be responsible for the higher adsorption capacities registered for the SH series of composite beads [54].

**Figure 6:**
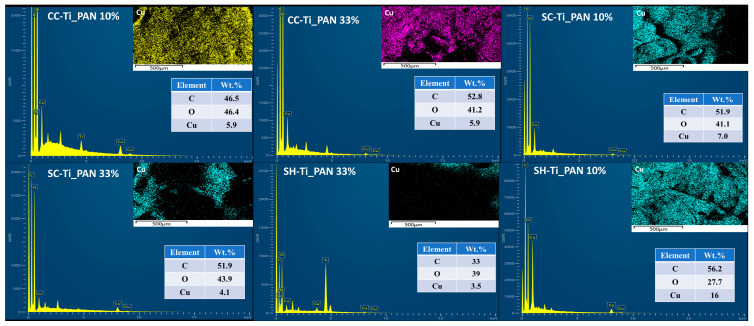
EDX micrographs and distribution map of adsorbed Cu^2+^ ions on the surface of the series of commercial chitosan (CC-Ti_PAN 10%, CC-Ti_PAN 33%), commercial chitin-derived chitosan (SC-Ti_PAN 10%, SC-Ti_PAN 33%) and chitosan obtained from shrimp wastes (SH-Ti_PAN 10%, SH-Ti_PAN 33%) beads.

Furthermore, using SEM microscopy, elemental mapping and EDX spectra were collected for all series of chitosan-based composite beads after Cu^2+^ ion adsorption. As shown in Figure 6, EDX spectra confirm the presence of the major constituents of chitosan, carbon and oxygen. In addition, significant amounts of copper were found on the surface of polymeric beads, with percentages ranging between 3.5 and 16%. The percentage of Cu^2+^ on the surface of the polymeric beads varies according to both the type of chitosan used and the percentage of composite in the sample. The lowest percentage on the surface of 3.5% is found in the case of SH-Ti_PAN 33%. The percentages for commercial chitosan samples are similar (5.9%); for samples based on commercial chitin-derived chitosan, the ratios are 4.1% for SC-Ti_PAN 33% and 7% for SC-Ti_PAN 10%. Interestingly, the highest percentage of 16% was recorded for the system SH-Ti_PAN 10% beads, which seems to confirm the results obtained previously by the adsorption kinetics study.

## 3. Materials and Methods

### 3.1. Materials 

Commercial chitosan with a degree of deacetylation ≥75% (CC, Sigma Aldrich-St Louis, MO, USA) was used without further purification. Commercial chitin-derived chitosan with a degree of deacetylation ≥78 (SC), and chitosan obtained from shrimp shell (SH) with a degree of deacetylation ≥75 were synthesized in the laboratory by a previously reported method [42]. Glacial acetic acid (CHIMREACTIV SRL-Bucharest, Romania) was used to prepare 1%, 3% and 10% solutions in distilled water. A coagulation bath of 5% NaOH solution was prepared by dissolving NaOH (CHIMREACTIV SRL-Bucharest, Romania) in distilled water).

Inorganic–organic polymer composites were synthesized by the host–guest method, starting from mesoporous titania (TiO_2_) and acrylonitrile (AN), by a previously reported process [22]. The two steps of the process included the ultrasound-assisted impregnation/adsorption of AN into TiO_2_ pores in the first phase and the ultrasound-assisted polymerization of AN in the second phase [22]. 

Copper (II) acetate monohydrate (≥98%), purchased from J.T. Baker (Center Vally, PA, USA), was used for adsorption tests. All reagents, described further, used for the quantitative determination of copper were of analytical purity. Standard solutions of Certipur, 1000 mg/L and 100 mg/L, respectively, from Merk (Darmstadt, Germany) were used to draw a calibration curve 65% solution (*w*/*w*) of nitric acid from Scharlau and ultrapure water produced by Milli-Q, Integral System (Merk) with a resistivity of 18.2 MΩ/cm to prepare the standard solutions and samples. The purge gas for the ICP-OES was Argon 5.0 of 99.999% purity (Messer Romania Gaz SRL).

### 3.2. Synthesis of Composite Beads

To prepare chitosan-based polymer composite beads with inorganic–organic content, several steps were necessary. Firstly, 2% chitosan was dissolved in acetic acid solution under magnetic stirring (800 rpm) at a temperature of 60 °C for 5 h. Depending on the type of chitosan used, the concentration of acetic acid solution was 1% in the case of commercial chitosan (CC), 3% in the case of commercial chitin-derived chitosan (SC) and 10% in the case of chitosan obtained from shrimp shell wastes (SH). In the second step, after complete dissolution of the biopolymer and the formation of the gel, a 33% (wt. relative to the chitosan mass) or 10% (wt. relative to the chitosan mass) ratio of inorganic–organic composite based on mesoporous titanium dioxide and acrylonitrile was added. After complete homogenization, the warm solution was added into a syringe and dripped from 80 cm into a coagulation bath containing the 5% NaOH solution, under gentle magnetic stirring (50 rpm). The composite beads were left in the coagulation bath for 12 h for complete bead formation. After coagulation, the beads were washed several times with distilled water to neutral pH. The diameter of the beads was in the range of 4–5 mm. The last step of the process was freezing the samples at −20 °C followed by freeze-drying (at −70 °C). The notation of samples based on the three types of chitosan, and the percentage of inorganic–organic composite used in bead synthesis is summarized in Table 4. 

### 3.3. Characterization Techniques

Fourier-Transform Infrared (FTIR) spectra of the samples were recorded on a Nicolet™ Summit PRO FTIR Spectrometer (Thermo Fisher Scientific, Waltham, MA, USA) by acquiring 16 scans with a 4 cm^−1^ resolution in the 4000–500 cm^−1^ region.

X-ray diffraction (XRD) patterns were collected with a SmartLab (Rigaku Wilmington, MA, USA) equipment, operated at 45 kV and 200 mA, with Cu Kα radiation (wavelength λ = 0.1541 nm) in a parallel-beam configuration (2θ/θ scan mode). The scanned range was 2θ = 2–70°, with a scan rate of 8°/min. 

The Brunauer–Emmett–Teller (BET) and Barrett–Joyner–Halenda (BJH) Methods. BET surface area and pore measurements for raw and obtained materials were carried out by nitrogen adsorption using a NOVA 2200 analyzer (Quantachrome Instruments, Odelzhausen, Germany). Nitrogen sorption isotherms at −196 °C were recorded after all the samples were outgassed at 40 °C for 4 h under vacuum prior to N_2_ adsorption.

Scanning electron microscopy (SEM) images of the polymeric composite beads were obtained using a TM4000 plus II product by (Hitachi, Krefeld, Germany) scanning electron microscope equipped with a secondary electron (SE) detector at an acceleration voltage of 15 kV. Energy-dispersive X-ray spectroscopy (EDS) was used for spectrum recording and elemental mapping of samples with an Oxford Instruments (High Wycombe, UK) equipment. SEM images were recorded on wet beads after rigorous washing while mapping and EDX analysis were recorded on freeze-dried beads after the adsorption process.

### 3.4. Adsorption Study

In order to determine the heavy metal ion (Cu^2+^) concentrations, the Optima 2100 DV ICP-OES System (Perkin Elmer, Waltham, MA, USA) was used, a dual-view optical system with axial and radial views of the plasma in a single working sequence, operating with an independent transistorized radio frequency generator (40 MHz), used to determine Cu^2+^ ion traces. The nebulizer system is equipped with a PEEK Mira Mist^®^ (Perkin Elmer, Waltham, MA, USA) nebulizer coupled with a spray chamber—Baffled Cyclonic. The spectrometer consists of an optical module comprising an Echelle monochromator with a two-dimensional charged coupled device (CCD) detector, and a spectral range 165–800 nm. For the determination of copper in liquid samples (aqueous solutions) on the calibration slide with a concentration range of 1–10 mg/L, the dilution factor used was 1:25. Samples thus diluted were acidified with 0.5 mL of 65% nitric acid solution.

To evaluate the adsorption kinetics of the polymer composite beads, the following process was considered. The Cu^2+^ solution was prepared in distilled water (100 mg/L) and the solution was brought to pH 5. The experiments were carried out in 15 mL tubes, in which solutions were prepared with a liquid solid ratio, R_S/L_ = 1.5 mg/mL. The term R_S/L_ refers to the amount of material used in relation to the amount of solution, expressed in mg sample/mL solution. The tubes were closed to minimize evaporation of the solution and placed in a shaker (BENCHMARK SCIENTIFIC H5000-HC Multitherm Shake) at a stirring speed of 200 rpm. The beads were left in contact with the Cu^2+^ solution for a very specific period (2 h, 4 h, 6 h, 8 h, 16 h, and 24 h). After the time had elapsed, the solution was filtered and placed in a bottle with a silicon cap for and the following analysis. 

Quantification of the equilibrium adsorption process was performed by calculating the removal efficiency (%) (Equation (1)) and the adsorption capacity for metal ions (*q*, mg/g) (Equation (2)), knowing the initial (*C*_0_, mg/L) and the final (*C_f_*, mg/L) concentrations of metal ions, the solution volume (*V*, L) and the adsorbent mass (m, g).

(1) $$Removal\;Efficiency\;(\%)=\frac{{(C}_{0}-C_{f})}{C_{0}}\;\times \;100$$

(2) $$q=\frac{{(C}_{0}-C_{f})\times V}{m}$$

The obtained adsorption data were evaluated based on three distinct kinetic models, pseudo-second-order (PSO) kinetic model, the Elovich kinetic model and the intraparticle diffusion model. To fit the kinetic models, the adsorption capacity after 1440 min was used as the equilibrium adsorption capacity, *q_e._* The equations of the kinetic models used are shown below:

The pseudo-second-order (PSO) kinetic model:

(3) $$q_{t}=\frac{k_{2}q_{e}^{2}t}{1+k_{2}q_{e}^{2}t}$$

where *q_e_* (mg/g) is the adsorbate amount adsorbed at equilibrium, *t* (min) is time and *k*_2_ (g/mg·min) is the rate constant. 

The Elovich kinetic model:

(4) $$q_{t}=\beta ln\left(\alpha \beta t\right)$$

where *β* (g/mg) is the desorption constant of Elovich and *α* (mg/g min) is the rate constant. 

The intraparticle diffusion (ID) model:

(5) qt=kdiff×t12+C

where *k_diff_* (mg/g min^1/2^) is the rate constant and *C* is the parameter related to the layer thickness of the boundary. 

## 4. Conclusions

The present study was carried out to evaluate the efficacy of new types of composite adsorbents, based on different chitosan and inorganic–organic composite fillers for the efficient removal of Cu^2+^ ions from simulated waters. Comprehensive research on the adsorption capacity of the materials was undertaken, and composite polymer beads based on three types of chitosan were developed: commercial chitosan, commercial chitin-derived chitosan and chitosan obtained from shrimp wastes enhanced with CaCO_3,_ and a titanium oxide–polyacrylonitrile composite at different percentages. Polymer composite beads were evaluated in terms of morphology, physicochemical characteristics, and adsorption capacity.

FTIR spectroscopy confirmed that the TiO_2_–polyacrilonitrile composite was incorporated into the biopolymer matrix; the presence of the composite in the structure of the materials being attested by the band at 2242 cm^−1^, characteristic of the C-N group in the polyacrylonitrile structure. XRD patterns showed an anatase-type crystal structure of TiO_2_ and an ordered crystal structure of chitosan. BET analysis revealed a higher porosity, with a larger surface area and pore volume for the SH-Ti_PAN 10% beads compared to the other studied samples, which was confirmed by SEM analysis, indicating a roughness due to the presence of the composite in granular form on the entire surface of the beads.

The adsorption capacity of the beads was tested at pH 5 in a simulated solution with Cu^2+^ ions at a concentration of 100 mg/L, and the adsorption kinetic parameters (90–1440 min) were studied over time using three kinetic models (PSO, Elovich and ID). The results showed that the kinetic model that best fitted the obtained experimental data was the PSO model, suggesting limited surface adsorption and metal–ligand bond formation. Furthermore, the adsorption study showed that the type of chitosan used and the filler percentage significantly influence the adsorption capacity of the beads, the maximum capacity of 19.2 mg/g being registered for the SHC-Ti_PAN 10% sample. The adsorption properties of composite beads were mostly influenced by the type of chitosan used, judging from the small variations in the adsorption capacity and the retention efficiency between samples with the same type of chitosan. For instance, the removal efficiency for commercial chitosan-based composites varied between 29.5 and 29.9%; for commercial chitin derived-chitosan, the variation was between 32.5 and 34.0%; for chitosan obtained from shrimp shell waste, the variation fluctuated was between 49.6 and 51.7%. In support of these adsorption results, EDX mapping highlighted the presence of increased Cu^2+^ ions on the surface of the beads, particularly for SHC-Ti_PAN 10%.

As a concluding remark, the composite beads obtained in this study can serve as potential adsorbents of Cu^2+^ in wastewater treatment. Unlike powder adsorbents, the composite beads can be easily recovered after treatment by simple filtration procedures.

## Figures and Tables

**Table 1:** BET surface area and pore analysis of the polymeric beads.

Sample	BET Surface Area (m^2^ g^−1^)	Pore Surface Area (m^2^ g^−1^)	Maximum Pore Diameter ^1^ (nm)	Micropore Volume ^2^ (cm^3^ g^−1^)
CC-Ti_PAN 33%	1.108	1.880	4.343	0.002
CC-Ti_PAN 10%	1.260	1.911	4.543	0.002
SC-Ti_PAN 33%	0.357	2.415	4.343	0.002
SC-Ti_PAN 10%	0.977	1.725	4.152	0.002
SH-Ti_PAN 33%	2.401	2.994	4.543	0.004
SH-Ti_PAN 10%	3.334	9.443	4.543	0.011

^1^ Calculated by BJH method. ^2^ Measured at P/P0 = 0.99.

**Table 2:** Comparison of maximum adsorption capacities of various adsorbents for Cu^2+^.

Sample	Adsorption Capacity, q (mg/g) after 24 h	Retention Efficiency after 24 h (%)	Cu^2+^ Initial Concentration	Ref.
CC-Ti_PAN 33%	10.7	29.9	100	This study
CC-Ti_PAN 10%	10.1	29.5	100	This study
SC-Ti_PAN 33%	10.7	32.5	100	This study
SC-Ti_PAN 10%	11.1	34.0	100	This study
SH-Ti_PAN 33%	15.5	49.6	100	This study
SH-Ti_PAN 10%	17.0	51.7	100	This study
Chitosan-coated sand (CCS)	1.2	99.77	100	[44]
Modified chitosan transparent thin membrane (MCTTM)	8.5	-	25	[45]
Pine sawdust	1.7	-	300	[46]

**Table 3:** Kinetic parameters for Cu^2+^ adsorption for chitosan-based polymer beads, at R_S/L_ = 1.5 mg/mL, pH = 5, initial Cu^2+^ concentration of 100 mg/L, and time range 0–1440 min.

Kinetic Model	Parameters	CC-Ti_PAN 33%	CC-Ti_PAN 10%	SC-Ti_PAN 33%	SC-Ti_PAN 10%	SH-Ti_PAN 33%	SH-Ti_PAN 10%
Experimental data	*q_e_*_, exp_ (mg/g)	10.714	10.128	10.687	11.124	15.521	17.039
PSO	*q_e_*_2_^2^/(mg/g) k_2_/(g/(mg·min) R^2^	12.053 3.889 0.973	13.202 2.788 0.951	18.232 5.545 0.995	16.385 8.053 0.947	18.575 1.068 0.898	19.180 5.284 0.989
Elovich	α (mg/(g·min) β (g/mg) R^2^	0.114 0.385 0.970	0.074 0.307 0.921	0.021 0.167 0.959	0.025 0.190 0.949	0.049 0.192 0.910	1.196 0.345 0.975
ID	k_d_ (mg/g·min^1/2^) C (mg/g) R^2^	0.275 1.351 0.902	0.298 0.925 0.847	0.275 9.900 0.958	0.282 9.890 0.931	0.373 2.284 0.922	0.437 4.488 0.772

**Table 4:** Sample preparation.

Sample Code	Chitosan Type (Abbreviation)	Inorganic–Organic Composite, Ti_PAN (wt. %)
CC-Ti_PAN 33%	Commercial chitosan (CC)	33
CC-Ti_PAN 10%	Commercial chitosan (CC)	10
SC-Ti_PAN 33%	Chitosan from commercial chitin (SC)	33
SC-Ti_PAN 10%	Chitosan from commercial chitin (SC)	10
SH-Ti_PAN 33%	Chitosan from shrimp shell (SH)	33
SH-Ti_PAN 10%	Chitosan from shrimp shell (SH)	10

## Data Availability

All the data are available from the corresponding author, Anita-Laura Chiriac, email: anita-laura.radu@icechim.ro.
